# The master molecule that built biology: How water shaped the chemistry of life

**DOI:** 10.1002/pro.70532

**Published:** 2026-04-09

**Authors:** Juliana DiGiacomo, George D. Rose, Loren Dean Williams

**Affiliations:** ^1^ School of Chemistry and Biochemistry, Georgia Institute of Technology Atlanta Georgia USA; ^2^ NASA Center for Integration of the Origins of Life Georgia Institute of Technology Atlanta Georgia USA; ^3^ Jenkins Department of Biophysics Johns Hopkins University Baltimore Maryland USA

**Keywords:** biological polymers, chemical evolution, hydrogen bonding, hydrophobic effect, origins of life, prebiotic chemistry, wet‐dry cycles

## Abstract

The deep entanglement of biomolecular structure and function with aqueous systems supports the view that water actively sculpted both molecules and processes during the origins of life and continues to constrain evolution today. Nature's rules of biochemistry and biophysics have survived for nearly 4 billion years. The in vivo roles of water, the architectures of biopolymer backbones and side chains, the structure and function of ribose, ATP, the translation system, the genetic code, and certain inorganic cations persist unchanged. Hydrogen bonding and the hydrophobic effect predate life on Earth altogether. Here we examine fundamental forces that established, shaped, and continue to constrain biochemistry and biophysics. The results support a model in which life emerged through water‐based selection among diverse molecules and molecular ensembles, with molecular fitness defined by behaviors in and interactions with water. Life is thus composed of molecules that cooperate with, resist, and exploit the unique properties of water. Biological molecules employ chemical strategies that enable selective and controlled persistence in aqueous environments, a phenomenon we classify as recalcitrance. Molecular assembly reduces conformational heterogeneity, constrains dynamics, and sterically excludes reactive agents, including water and hydrolytic enzymes. By this mechanism, lifetimes of folded proteins, structured RNAs, assembled phospholipids, and polysaccharides are mediated by their organizational states.

## 1 INTRODUCTION

Water and life are fully entangled. While it might appear that water's properties are finely tuned to support life, the reverse is true: water predated life, and life emerged and evolved in response to water's unique physical and chemical characteristics. The causality runs from water to biochemistry, not the reverse.

Here we suggest that water shaped the chemical landscape on which life and its origins can be understood. During life's emergence, water enabled diverse possibilities and imposed powerful constraints. The molecular architectures that emerged and persisted, including phosphodiesters, peptides, and amphiphiles, are responses to water's physicochemical imperatives.

Only certain molecular species and reaction networks could emerge and evolve in the context of water. Consequently, biology is composed of molecules that exploit, accommodate, and resist water's unique properties. These roles are not frozen accidents but instead reflect the results of chemical evolution. Dehydration–condensation, hydrolysis, the hydrophobic effect, amphoterism, and aqueous ion chemistry were not impediments that evolution overcame but channels that guided chemical evolution from the onset.

**FIGURE 1:**
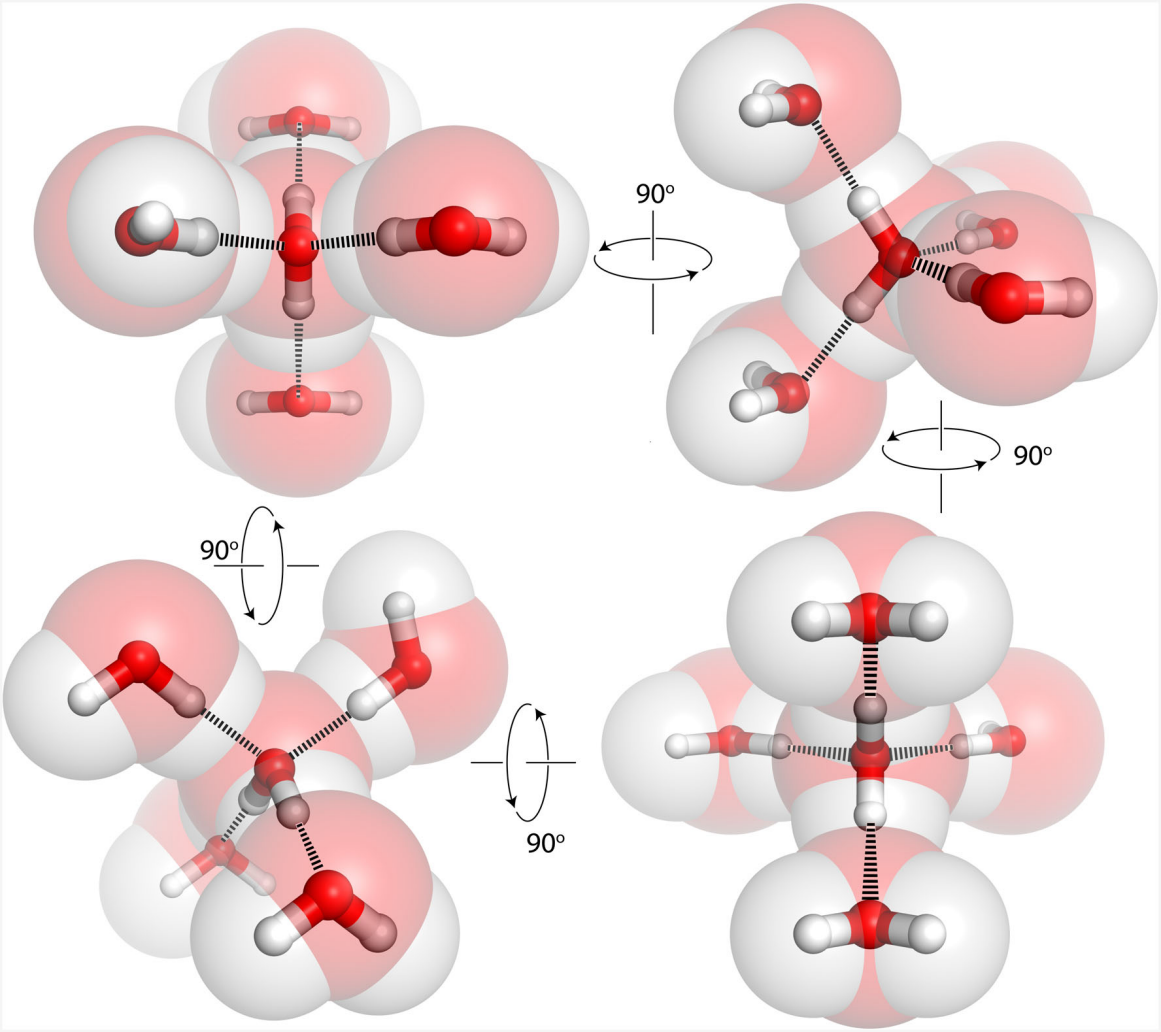
In the condensed state (solid or liquid), a water molecule, with pseudo‐tetrahedral point symmetry, forms cohesive interactions (hydrogen bonds) with other water molecules in pseudo‐tetrahedral space symmetry.

Some models of prebiotic chemistry treat water as a passive medium or as a problem to be solved (Benner, 2014; Orgel, 1998; Powner et al., 2009; Shapiro, 1986). We argue the inverse: Water acted on and selected organic molecules. Naturally occurring processes, such as wet/dry cycling, created and selected polymers under conditions where water's alternating presence and absence imposed competing pressures. The backbone chemistries that arose from this selection have persisted for 3.5 billion years, not because they were optimal, but because they represented immediate responses to water's chemical imperatives in the lead up to life. In the following sections, we examine how water (Figure 1) governed molecular behavior, guided biological folding and assembly, and constrained evolutionary possibilities. Water established, shaped, and continues to govern biochemistry and biophysics.

The roles of water at the core of biochemistry have remained invariant across the tree of life, from the last universal common ancestor to the present, and from bacteria to archaea and eukarya. For nearly 4 billion years, water has been the dominant physical medium of biology—the primary bulk phase in which biochemical reactions occur, and is the major constituent of living matter by mass (Ball, 2017; Frenkel‐Pinter et al., 2021; Milo & Phillips, 2015). All of biology depends on the aqueous coordination of metal cations such as Na^+^, K^+^, Mg^2+^, Ca^2+^, and Zn^2+^, whose hydration shells determine effective size, charge distribution, and reactivity (Lippard & Berg, 1994). Nowhere do we find biology that forms peptide, phosphodiester, or glycosidic bonds by mechanisms other than dehydration condensation (Frenkel‐Pinter et al., 2021; Nelson et al., 2021). Everywhere in biology, energy transduction depends on water as a nucleophile—ATP hydrolysis, GTP hydrolysis, and phosphoryl transfer all exploit water's reactivity to break high‐energy bonds (Nelson et al., 2021). Everywhere in biology we find membranes stabilized by the hydrophobic effect (Nelson et al., 2021). Nowhere in biology do we find buffering, acid–base homeostasis, or redox equilibria independent of water's amphoteric, dielectric, and hydration properties, which define proton mobility, pH and pKa scales, and redox reference potentials.

Water, through the hydrophobic effect, uniquely drives protein folding (Baldwin & Rose, 2016; Rose et al., 2006), and nucleic acid assembly. Biopolymers spontaneously adopt highly ordered conformations with low configurational entropy. Water stabilizes specific states while excluding others. Enzymes contain well‐defined hydrophobic interiors, hydrophilic exteriors, and catalytic clefts that exclude or specifically localize water (Fersht, 1985; Kauzmann, 1959; Lee et al., 2007). Water stabilizes transition states in enzymatic reactions by organizing electrostatic fields, mediating proton transfer, and forming transient hydrogen bonds (Ball, 2008). Water has endowed the Earth with dissolved salts and electrolytes (Wright, 2007), compartmentalization (Menon et al., 2017), and phase separation (Hatters, 2023). Water's context‐dependent effects include on‐water versus in‐water catalysis (Butler & Coyne, 2010) and a distinction between dilute solutions and high‐solids/low‐water matrices (Slade et al., 1991).

Water is at once commonplace and strange. It is everywhere in daily life, condensing on cold beer cans, forming clouds, rain, lakes, and oceans, and sustaining all known life. It covers most of Earth's surface. Water is the third most abundant molecule in the universe, after H_2_ and CO (Ceccarelli, 2020; Omont, 2007). It is deeply embedded in chemistry, biology, ecology, culture, and the economy. This everyday familiarity obscures physical and chemical properties that are profoundly unusual—unlike those of any other known substance.

## 2 MOLECULAR WATER

**FIGURE 2:**
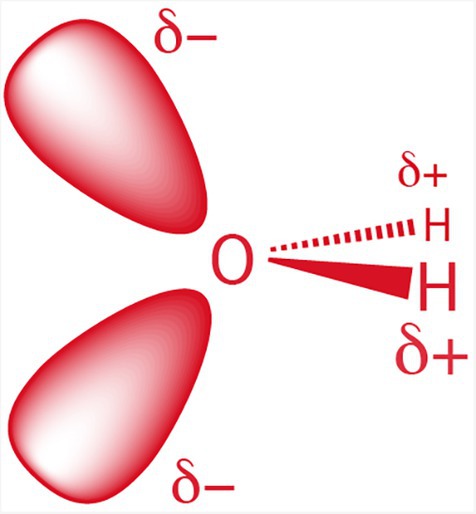
A water molecule has two bonding orbitals and two non‐bonding orbitals.

The unusual properties of liquid water originate in the structure of the water molecule (Figure 2), which drives formation of dense and extended hydrogen bonding networks in condensed phases (Figure 1). Water is small and polar with C_2V_ symmetry, meaning it has a single two‐fold rotation axis and two perpendicular mirrors. The pseudo‐symmetry of a water molecule is *T*_d_ (tetrahedral), and this point symmetry conforms to the space pseudo‐symmetry of solid and liquid water. The local symmetry of an individual water molecule aligns with the symmetry of its surroundings in the condensed state, allowing each water molecule to integrate seamlessly into liquid and solid structures (Figure 1). This match in symmetries allows the basic structure of the condensed phase to be maintained during rotations of water molecules. Before and after rotation, an individual water molecule (Figure 2) can maintain four hydrogen bonds. The interplay of cooperative intermolecular forces, geometric complementarity, and bonding multiplicity endows water with the remarkable capacity to form dense and dynamic three‐dimensional networks of hydrogen bonds.

### 2.1 Hydrogen‐bonding

**FIGURE 3:**
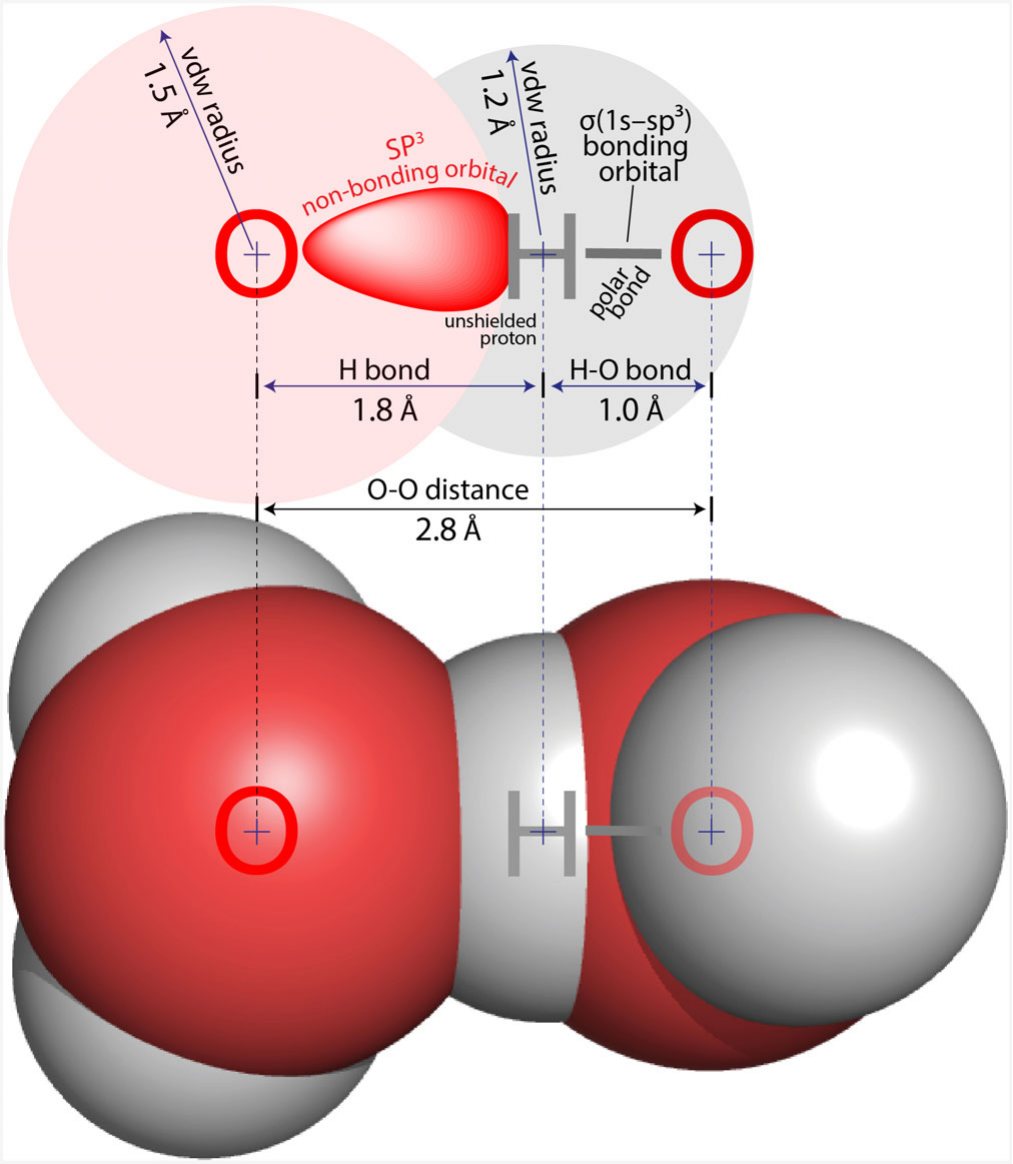
In a water–water hydrogen bond, a hydrogen nucleus from one water molecule is donated to an sp^3^ lone pair on the oxygen atom of a neighboring water molecule, along the axis of the O–H covalent bond. The polar O–H bond withdraws electron density from the hydrogen atom, exposing the back of the proton to HB acceptor.

Hydrogen bonds are attractive forces between deshielded hydrogen atoms and basic lone pairs of electrons (Figure 3). Hydrogen is unique in that it forms polar covalent bonds with electronegative atoms using its 1s electron, thereby exposing its nucleus (Pimentel & Mcclellan, 1971). The resulting deshielded, cationic face of the proton attracts the partial negative charge of a lone pair on an adjacent molecule. Other atoms that form covalent bonds with electronegative elements possess inner‐shell nonbonding electrons that shield their nuclei and preclude comparable interactions.

A hydrogen bond is primarily electrostatic. However, partial covalency in hydrogen bonding arises from orbital overlap, which becomes more significant as the hydrogen bond angle approaches linearity and the length decreases (Iogansen, 1999). The balance of electrostatic and covalent contributions to hydrogen bonding has yet to be fully resolved (Jeffrey & Saenger, 2012).

ven a single hydrogen bond can tip the scales of folding thermodynamics. Hydrogen bond enthalpy values in the vapor phase range from −5.7 kcal/mol between water molecules to as much as −20 kcal/mol when one partner is charged (fig. 1 in Rose & Wolfenden (1993)). Typical values in biological systems, such as those within protein secondary structure or between paired nucleobases, are at the lower end of this range, around −5 kcal/mol. The enthalpy of an intramolecular peptide hydrogen bond is slightly more favorable than that of an intermolecular hydrogen bond with water, by around 1.0 kcal/mol at physiological temperature (Scholtz et al., 1991). However, these small energy increments are sufficient to shape thermodynamic landscapes of biological systems.

The exact geometry of hydrogen bonding (i.e., distance and angle dependence) is understood from highly accurate small‐molecule crystal structures (Taylor et al., 1983) or from large databases of macromolecular structures and simulations (Baker & Hubbard, 1984; Hagler et al., 1974; Hagler & Lifson, 1974; Jeffrey & Saenger, 2012; Stickle et al., 1992).

### 2.2 Hydrogen bond networks

Hydrogen bonds are, of course, ubiquitous in organic systems and minerals. However, the density of hydrogen bonds in liquid and solid water is unmatched by any other known substance (Table 1). No other molecule achieves such a high number of cooperative cohesive interactions per unit volume, making water uniquely capable of forming continuous, three‐dimensional hydrogen‐bond networks. At this density, bonding interactions among molecules are effectively continuous, confering solid and liquid water with a high level of structural connectivity. A combination of high hydrogen‐bond density and dynamic rearrangement enables water to mediate chemical interactions. Dense, geometrically optimized, dynamic, and cooperative hydrogen‐bond networks underlie water's unusual dielectric constant, density, heat capacity, pH buffering capacity, nucleophile and electrophile activation, and the hydrophobic effect (Ball, 2017; Brack, 1993; Brini et al., 2017; Finney, 2004).

**TABLE 1:** Hydrogen bond density in liquids and minerals.^a^

Liquid	Molecules per mL (× 10^22^)^b^	Unique H‐bonds per molecule	H‐bond density per mL (× 10^22^)
Water (liq) (Guardia et al., 2015)	3.34	2.0	6.68
Hydrogen fluoride (Orabi & Faraldo‐Gómez, 2020)	2.98	1.1	3.28
Formamide (Ozkanlar, 2018)	1.51	1.7	2.65
Ammonia (Krishnamoorthy et al., 2022)	2.41	0.75	1.81
Hydrogen sulfide (Sarkar & Bandyopadhyay, 2019)	1.6	0.8	1.28
Formic acid (Senthilkumar et al., 2006)	1.60	1.0	1.60
Glycerol	0.84	1.75	1.47
Methanol (Bellissima et al., 2016)	1.49	0.85	1.27
Mineral (Klein & Dutrow, 2007)^c^			
Water (solid)	3.07	2.0	6.13
Epsomite MgSO_4_·7H_2_O	0.41	14	5.75
Gibbsite Al(OH)_3_	1.87	3	5.61
Mirabilite Na_2_SO_4_·10H_2_O	0.28	20	5.57
Brucite Mg(OH)_2_	2.47	2	4.94
Gypsum CaSO_4_·2H_2_O	0.81	4	3.25
Boehmite.AlO(OH)	3.02	1	3.02
Goethite FeO(OH)	2.89	1	2.89
Kaolinite Al_2_Si_2_O_5_(OH)_4_	0.61	4	2.43
Natrolite Na_2_Al_2_Si_3_O_10_·2H_2_O	0.52	4	2.07

^a^ This table indicates the number of ideal hydrogen bonds at low temperature. For all substances, the extent of ideality of hydrogen bonds decreases with temperature.

^b^ A molecule is indicated by a formula unit (e.g., MgSO_4_·7H_2_O is counted as one molecule).

^c^ Ice is considered to be a mineral.

*Dielectric effects*. Water has a remarkably high dielectric constant and an exceptional ability to dissolve charged and polar molecules. At room temperature, the dielectric constant of water is approximately 80, greatly attenuating electrostatic interactions, allowing salts and other electrolytes to dissociate and remain solvated. A tin spoon filled with table salt (NaCl) over a Bunsen burner will melt before the salt. Empty the spoon into a glass of water, and the ions separate readily. The weakening of Coulombic forces by water lowers the energetic penalty for close like‐charges, for example, between anionic phosphates in nucleic acids. Delocalized counterions accumulate and partially neutralize highly charged macromolecules, reducing the effective electrostatic potential and stabilizing the system (Lipfert et al., 2014; Manning, 2002). This phenomenon makes the folding of nucleic acids possible while maintaining repulsion, allowing strand separation and unfolding.

*Density*. Water is one of the few substances that expands upon freezing. The molar volume of liquid water is about 8% less than that of ice, reaching a minimum at 4°C and increasing only slightly with rising temperature. The probability and lifetime of ideal tetrahedral hydrogen‐bonded configurations increase as the temperature decreases from 4 to 0°C. Such configurations occupy a larger volume (lower density) than at higher temperatures, where non‐ideal hydrogen bonding can favor molecular compaction. This characteristic, together with water's high heat capacity, insulates aquatic habitats and preserves the liquid state even when ambient temperatures fall below freezing, allowing life to persist in extreme conditions.

Among the many striking behaviors of water is the regelation of ice, demonstrated in Faraday's classic experiment (Faraday, 1860). A taut wire, weighted at both ends, gradually passes through a block of ice—while the block itself remains intact. The pressure exerted by the wire lowers the melting point of the ice directly beneath it, causing localized melting; as the wire advances, the water refreezes behind it. Unlike most substances, the solid–liquid equilibrium line of water slopes negatively in a pressure–temperature phase diagram, meaning that the application of pressure lowers the melting point of ice instead of raising it (as would be the case for most solids). This phenomenon is what allows glaciers to slide over bedrock (Alley et al., 2019).

*Heat capacity*. Liquid water has a high specific heat capacity, meaning that large quantities of absorbed energy produce only small changes in temperature. Much of this energy is taken up by rearrangements within the hydrogen‐bond network before appearing as translational, rotational, or vibrational motion (Brewer & Peltzer, 2019). As such, water buffers thermal fluctuations across multiple scales. At the cellular level, water serves as an energy buffer, absorbing or releasing heat while maintaining a relatively constant temperature. Water moderates temperature changes at the level of ecosystems, stabilizing aquatic environments. On a planetary scale, the heat capacity of seawater absorbs seasonal heat variations, moderating weather and climate.

*Amphoterism*. Water is amphoteric, meaning it can act as both a proton donor and a proton acceptor. It undergoes autoprotolysis to produce hydronium and hydroxide ions, establishing the neutral point of the pH scale and participating in proton‐dependent reactions. Proton mobility through hydrogen‐bonded networks occurs via Grotthuss shuttling (Knight & Voth, 2012), which enables rapid proton transport in channels and proton‐coupled energy transduction, thereby tightly linking water structure to bioenergetics (Hassanali et al., 2013). Amphoterism is fundamental to enzyme catalysis, proton transport, and metabolic reactions that depend on precise control of proton flux.

*Reactivity*. Water governs both the kinetics and equilibria of biological reactions and defines much of life's reactivity landscape. Most biological molecules are synthesized by condensation–dehydration reactions and degraded by hydrolysis (Frenkel‐Pinter et al., 2021). In hydrolysis, water (or hydroxide derived from it) acts as a nucleophile that attacks electrophilic centers such as carbonyl or phosphate groups. In condensation, the reverse process, bond formation is accompanied by the release of water. Water solvates nucleophiles, electrophiles, and charged intermediates, lowering activation barriers and rendering otherwise inaccessible reactions accessible (Phan & Mayr, 2005). In sum, the combined nucleophilic, amphoteric, and solvation properties of water make it the dominant reactive species and the principal agent of chemical transformation in biology.

*Specificity* versus *stability*. Folded and assembled states of biopolymers tend to minimize unsatisfied donors and acceptors and maximize intramolecular hydrogen bonds, within *α*‐helices, *β*‐sheets, and base pairs (Rose, 2021; Von Hippel & Berg, 1986). Yet, in water, intramolecular hydrogen bonds generally do not contribute much to the favorable free energy of the folded state. If intramolecular hydrogen bonds are disrupted by partial unfolding, they are simply replaced by water‐biopolymer hydrogen bonds. However, an unsatisfied hydrogen bond donor or acceptor within a folded or assembled biopolymer confers a net gain in hydrogen bonding during unfolding, and can be strongly destabilizing, by much as +5 kcal/mol.

Yet molecular recognition often depends on accessibility of hydrogen‐bonding groups in ligand binding sites of proteins or complementary strand recognition through base pairing. The delicate balance between satisfying hydrogen bonds for stability and allowing specific patterns of unsatisfied or exposed groups for recognition is driven by the unique strength, directionality, and reversibility of hydrogen bonds in water.

### 2.3 Hydrolysis and condensation—wet and dry

Considering its properties, water appears to present a paradox at the heart of biochemistry. Water spontaneously degrades biopolymers by hydrolysis yet contributes to their persistence by stabilizing folds and assemblies that resist hydrolysis.

The polymerization of building blocks to form DNA, RNA, polypeptides, or polysaccharides is thermodynamically unfavorable in aqueous media (Lindahl, 1993; Martin, 1998; Peller, 1976; Ross & Deamer, 2016; Westheimer, 1987; Wolfenden, 2006; Wolfenden et al., 1998). All universal biopolymers spontaneously hydrolyze in water. This inherent chemical instability of biopolymers has been termed the “water problem” (Benner, 2014; Orgel, 1998; Powner et al., 2009; Shapiro, 1986). Numerous models have been proposed to explain how monomers could form biopolymers spontaneously during the chemical phase of the origins of life. These models include chemical activation of building blocks (Ferris, 2006; Hill & Orgel, 2002; Prywes et al., 2016; Rabinowitz et al., 1969; Steinman et al., 1964), polymerization on mineral surfaces (Erastova et al., 2017; Hazen & Sverjensky, 2010; Orgel, 1998; Wächtershäuser, 1988), polymerization in hydrothermal environments (Imai et al., 1999), and polymerization via energy‐dissipative cycling reactions (Sutherland, 2017).

An alternative model, which we favor, assumes that both driving forces and kinetics of hydrolysis and condensation are state‐dependent (Deamer & Weber, 2010; Ross & Deamer, 2016, 2019). In this view, the thermodynamic driving force is governed by the chemical potential of water, which can vary widely across conditions. The driving force for hydrolysis or condensation reactions in dilute solutions can be roughly estimated based on water activity (Deamer & Weber, 2010; Ross & Deamer, 2016, 2019) (Table 2). This table is based on a condensation dehydration reaction given by:

(1) $$N_{p}+N⇌N_{p+1}+H_{2}O.$$

In Equation (1), *N* is a monomeric building block, *N*_*p*_ is a polymer of length *p*, and *N*_*p*+1_ is a polymer of length *p* + 1, formed by the loss of water in a condensation–dehydration reaction. The driving force for the reverse reaction, the hydrolysis of *N*_*p*+1_ back to *N*_*p*_ and *N*, is given by Equation (2), where $a_{N_{p}}$ is the activity of polymer *N*_*p*_, $a_{N}$ is the activity of building block *N*, $a_{N_{p+1}}$ is the activity of polymer *N*_*p+*1_, and $a_{w}$ is the activity of water. Equation (3) resolves concentrations and activity coefficients, γ, which indicate deviation from ideal behavior. In ideal solutions, each activity coefficient one. A net γ correction, modifies the concentration reaction quotient, as displayed in Equation (4).

(2) $$\Delta G=\Delta G^{o}+\text{RTln}\frac{a_{N_{p+1}}\;}{a_{N_{p}}*a_{N}}a_{w}$$

(3) $$\Delta G=\Delta G^{o}+\text{RTln}\frac{\gamma_{N_{p+1}}\left[N_{p+1}\right]}{\gamma_{N_{p}}\left[N_{p}\right]*\gamma_{N}\left[N\right]}\gamma_{w}\left[H_{2}O\right]$$

(4) $$\Delta G=\Delta G^{o}+\text{RTln}Q+\text{RTln}\frac{\gamma_{N_{p+1}}}{\gamma_{N_{p}}*\gamma_{N}}\gamma_{w}$$

**TABLE 2:** Estimated contributions to Gibbs free energy (Δ*G*) during wet–dry cycling.

	Contribution to Δ*G*			Δ*G* = Δ*G*°+ RTln*Q* + γ correction					
	RT ln Q kcal mol^−1^	RT ln Q kcal mol^−1^	γ correction kcal mol^−1^	Protein, Amide Δ*G*°= 2.19 kcal mol^−1^		Polysaccharide, glycoside Δ*G*°= 3.80 kcal mol^−1^		Nucleic acid, phosphate ester Δ*G*°= 3.29 kcal mol^−1^	
*T*, °C	(Wet)^a^	(Dry)^b^	(Dry)^c^	Wet	Dry	Wet	Dry	Wet	Dry
25	3.7	−1.0	−0.8	5.9	0.4	7.5	2.0	7.0	1.5
50	4.1	−1.1	−0.9	6.3	0.2	7.9	1.8	7.4	1.3
75	4.4	−1.2	−1.0	6.6	0.1	8.2	1.7	7.7	1.2
100	4.6	−1.2	−1.0	6.8	−0.1	8.4	1.5	7.9	1.0

*Note*: (Table adapted from Ross & Deamer, 2016). Standard biochemical Gibbs energies, taken from (Table 13–4 in Nelson et al., 2021)) based on the reactions: glycylglycine + H_2_O → 2 glycine, lactose + H_2_O → glucose + galactose, glucose‐6‐phosphate + H_2_O → glucose + *P*_*i*_.

^a^ *Q* is the reactant quotient. Wet state: [H_2_O] = 55.0 M, [*N*_*p*_] = [*N*_*p*+1_] = [*N*] = 0.10 M.

^b^ Dry state: [H_2_O] = 0.55 M, [*N*_*p*_] = [*N*_*p*+1_] = [*N*] = 3.00 M.

^c^ Dry state γ corrections: $\gamma_{w}\approx$ 0.5, $\gamma_{N_{p}}$ ≥ $\gamma_{N_{p+1}}$ ≈ $\gamma_{N}\approx$ 2.0.

During evaporation of an aqueous solution, water activity decreases, building block activities increase, and the solution becomes increasingly non‐ideal (Barbosa‐Cánovas et al., 2020; Held et al., 2011; Mauer et al., 2000). Equation (2) predicts that the free energy of condensation would decrease as a solution of building blocks is concentrated. As an exercise of this concept, we present Table 2, which estimates changes in free energies of condensation as water activity decreases 100‐fold and solute concentration increases. Dry state γ corrections are conceptual approximations in alignment with estimates of the impact cavity size and molecular packing from Ross and Deamer (2016).

However, food chemists and others have investigated dried systems that contain residual water, and have concluded that water activity does not accurately account for all observed behaviors in the dry realm (Slade et al., 1991). Here we use the term *high‐solids/low‐water matrix* to refer to a condensed phase dominated by solutes or solids, containing residual water, with restricted water mobility, and complex, multistate hydration. A high‐solids/low‐water matrix corresponds to a dry state in wet‐dry cycling reactions. Equation (2) cannot be used to quantitatively predict the driving force of condensation in the dry state; it does not apply to high‐solids/low‐water matrices. However, experimentally, it is observed amino acids do condense spontaneously to form peptides at reasonable rates in high‐solids/low‐water matrices (Forsythe et al., 2015; Frenkel‐Pinter et al., 2022; Matange, Rajaei, et al., 2025). Therefore, in high‐solids/low‐water matrices, thermodynamic driving forces and kinetic accessibility leads to condensation even though dynamics and conformational transitions are suppressed (Slade et al., 1991).

In sum, the “water problem” of spontaneous hydrolysis applies in one set of conditions but not others. Wet‐dry cycling causes oscillations from dilute aqueous solution to *high‐solids/low‐water matrix* and can drive oscillations in hydrolysis and condensation. The behavior and properties of water are not fixed but are emergent and contingent on chemical and physical environment. Net synthesis would prevail during wet‐dry cycling if the time constant of the wet phase is short and the rates of hydrolysis are slow. We propose that the dynamic balance between condensation and hydrolysis characteristic of biochemistry emerged from selection acting on chemical systems during the origins of life. Under this scenario, condensation products that withstood hydrolytic stress during recurrent wet–dry cycling on the Hadean Earth were selectively preserved and propagated.

The diverse chemical feedstocks characteristic of the Hadean Earth (Cody et al., 2000; Ehrenfreund & Sephton, 2006; Glavin et al., 2025; Parker et al., 2011; Zahnle et al., 2020) would have been continuously subjected to wet–dry cycling on land surfaces. From the Early Hadean Eon (~4.5–4.0 Ga) to the present day, atmospheric water activity has varied with the diurnal period, alternating between condensation and evaporation across terrestrial surfaces. Oceanic islands and other landforms that dotted the oceans throughout Earth's history, including during the Hadean (Korenaga, 2021), were regularly exposed to diurnal wet–dry cycles. More localized settings capable of such cycling include hot springs, evaporative pools replenished by precipitation, geyser splash zones on surrounding hot rocks, recurring tidal pools, and combinations of these environments (Campbell et al., 2019; Damer & Deamer, 2015, 2020).

### 2.4 Metal cations

The metal palette of biochemistry was determined in part by competition between water and silicate lattices for cations. Silicates dominate Earth's mantle and crust and sequester a subset of metals, rendering them unavailable to biology (Goldschmidt, 1954). Al^3+^, Ti^4+^, Zr^4+^, and the rare earth elements are strongly lithophilic and are locked into silicate minerals. Na^+^ and K^+^ are only weakly incorporated into silicates and therefore remain soluble in water. Mg^2+^ and Ca^2+^ partition into silicates and carbonates yet remain moderately soluble and are mobilized by weathering. Together, these four metals account for >99% of all metal cations in biology (Milo & Phillips, 2015).

Metal cation solubility in water reflects a balance between hydration enthalpy, the enthalpy change when water molecules coordinate a cation, and lattice enthalpy, the enthalpy change required to dissociate metals from a solid (Weller et al., 2025). Ions whose hydration enthalpies exceed their lattice enthalpies are water‐soluble. This balance predicts the observation that monovalent and divalent cations dominate biochemical systems, whereas many highly charged or highly polarizing cations form stable mineral phases with large lattice enthalpies and are absent from biology.

Biochemical metal cations can be fully hydrated as [M(H_2_O)_*n*_]^m+^ or partially hydrated as [M(H_2_O)_*n*_L_*m*_]^p+^. In cells, hydrated metal cations, especially K^+^, accumulate in dynamic, disordered layers near DNA, RNA, and membranes. This counterion condensation reduces the effective negative charge (Manning, 2002) and enables formation of DNA duplexes, folded RNAs, and membranes. In other environments, biochemical ligands displace water from the first coordination shell. Large folded RNAs are stabilized by Mg^2+^ ions, in which some first‐shell water molecules are replaced by phosphate oxygens through inner‐sphere coordination (Hsiao & Williams, 2009). Metal cations form bridges between uncharged ligands, as in zinc fingers in proteins (Krishna et al., 2003), or between charged groups, as in magnesium clamps in RNA (Hsiao & Williams, 2009).

The exchange of first‐shell water molecules with biomolecular ligands underlies much of metal selectivity and biological tuning of metal reactivity (Dudev & Lim, 2014). For Group 1 and 2 cations, ionic radius and charge density govern hydration structure and energetics. Mg^2+^, with its small radius, high charge density, and closed‐shell electronic configuration, behaves as a hard Lewis acid and forms a rigid, octahedral first hydration shell with a large hydration enthalpy. The geometry is dictated by Mg^2+^–O interactions and packing within the first coordination shell (Bowman et al., 2012). Ca^2+^ adopts a higher coordination number (typically 7–8 vs. 6 for Mg^2+^) with a more flexible and less constrained geometry. Interactions of water with transition‐metal cations acquire partial covalent character through σ‐donation from water lone pairs into metal d orbitals, leading to variable coordination numbers and geometries governed by ligand‐field effects, including Jahn–Teller distortions. First‐shell water molecules of biological metal cations exchange with water molecules of bulk water, generally on timescales from nanoseconds to microseconds, depending on the metal (Bleuzen et al., 1997; Helm & Merbach, 2005). Exchange of Mg^2+^ first‐shell water is slow (residence time ~10^−6^ s) relative to Ca^2+^ (~10^−9^ s) because of its higher charge density.

Cations dominate over anions in biochemical functions in part because they are more strongly hydrated. Ion–dipole interactions between cations and the δ^−^ oxygen of water are generally stronger than those between anions and the δ^+^ hydrogens. Anions are typically larger and have lower charge densities than comparable cations, which reduces their hydration enthalpies.

In sum, by governing both the availability and coordination behavior of metal ions, water defined the metal chemistry of the prebiotic environment and has preserved it in modern biochemistry.

## 3 FOLDING

During the origins of life, water was the primary selective agent of chemical evolution, determining the metal cations, small organic species, and polymers of biochemistry. In this manner, water selected for soluble ions and folding‐competent polymers. In this section we explain how water selected polymers with sophisticated folding proficiencies. Biopolymer folding arises from complementarity that can be cohesive (between like) or adhesive (between unlike) species and can be intermolecular or intramolecular. Folding can be driven in part by the hydrophobic effect and is modulated by Hofmeister‐like ions (Von Hippel & Schleich, 1969) and osmolytes (Bolen & Rose, 2008).

### 3.1 The hydrophobic effect

At first glance, the spontaneous demixing of oil and water appear to violate the second law of thermodynamics. This apparent paradox highlights the necessity to account for all entropic and enthalpic effects, including solvent entropy, during biopolymer folding in aqueous environments.

Walter Kauzmann (1959) and Tanford (1980) laid the groundwork for much of contemporary thinking about the hydrophobic effect. Kauzmann emphasized the thermodynamic driving force for transferring hydrocarbons from water to organic solvents, highlighting the unfavorable interactions between water and nonpolar solutes (tab. III in Kauzmann (1959)). Tanford (1962), by contrast, proposed that protein folding is largely driven by the expulsion of water from hydrophobic side chains. Many explanations for the hydrophobic effect have been proposed (Creighton, 1993; Dill, 1990; Graziano, 2015; Kauzmann, 1959; Lee, 1991; Tanford, 1980), though a complete mechanistic picture remains an active area of inquiry. What is clear is that the hydrophobic effect can play a major role in the folding of proteins (Anfinsen, 1973) by engendering solvent‐sequestered interiors that shift the unfolded ⇌ folded equilibrium to the right.

### 3.2 Recalcitrance: The protective blanket of folding

Universal biopolymers manipulate hydrolysis through folding, supporting models in which water was the primary selective agent during their emergence. By driving both folding and hydrolysis, water indirectly controls the thermodynamic and kinetic landscape of polymer degradation, a property known as recalcitrance. Recalcitrance was first identified by saccharide chemists (Beckham et al., 2011) and later recognized as a general property of all universal biopolymers (Edri et al., 2023; Guth‐Metzler et al., 2023; Matange, Marland, et al., 2025).

Recalcitrance is protection against hydrolysis that arises from folding‐reduced conformational heterogeneity, folding‐constrained molecular dynamics, and folding‐reduced steric access of reactive agents, including water, radicals, and hydrolytic enzymes (Edri et al., 2023; Guth‐Metzler et al., 2023; Matange, Marland, et al., 2025). Tertiary and secondary structure formation shields the protein backbone from solvent. Over 80% of the solvent‐accessible backbone surface of polyalanine is lost for central residues upon formation of an *α*‐helix (Marqusee et al., 1989). *β*‐sheet formation also reduces solvent‐accessible surface area (Yan et al., 2007; Zhang et al., 1994).

RNA is also protected from hydrolysis through folding (Guth‐Metzler et al., 2023), even though the folded RNA backbone often remains solvent‐exposed. This distinction highlights the importance of dynamics in determining reactivity. Single‐stranded RNAs are more reactive because high conformational dynamics increase the probability of occupying transition states. In contrast, more static RNAs, such as folded tRNAs and rRNAs, are less reactive because conformational rigidity decreases that probability. In addition, juxtaposed RNA helices can provide shielding from solvent access by sharing counterions (Murthy & Rose, 2000), which further confers resistance to hydrolysis. Extremes on the free‐energy landscape include amyloids and cellulose, which occupy deeply trapped, thermodynamically stable states, whereas globular proteins and folded RNAs reside in shallower wells.

**FIGURE 4:**
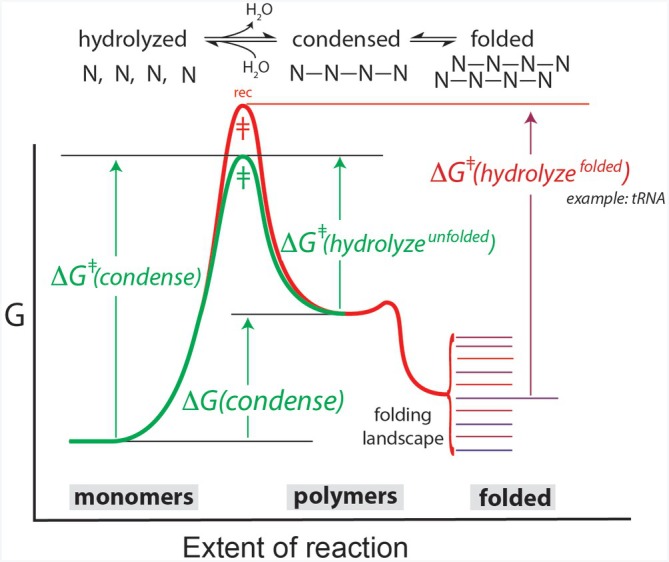
The fates of biological molecules in aqueous media are modulated by folding. This reaction free energy surface illustrates how both the activation and reaction free energies for hydrolysis are coupled to assembly. A given biological molecule can participate in a variety of folded states, each associated with a distinct hydrolysis free energy landscape.

The hydrolytic lifetimes of proteins, RNAs, polysaccharides, and even phospholipids are governed by their folding states. Reaction free‐energy landscapes are directly coupled to folding free‐energy landscapes, enabling modulation of lifetimes and chemical stabilities via non‐covalent molecular interactions. A given type of biopolymer can occupy many types of folded states, corresponding to many distinct topographies of the reaction free‐energy surface (Figure 4). The entire reaction free‐energy surface, including both catalytic activation and reaction free energies, is coupled to biopolymer folding. The simultaneous control of folding and reactive landscapes by water is strong evidence that biopolymers emerged via water‐driven chemical evolution.

### 3.3 Divergence and convergence

In organismal biology, mutualisms are characterized by distinct and complementary proficiencies. Molecular mutualisms in biochemistry also demonstrate complementarity of proficiencies (Lanier et al., 2017). RNA and proteins are related by distinct physical and chemical asymmetries. These two biopolymers present fundamentally different faces to the environment. The polypeptide backbone is conformationally constrained (Ramakrishnan & Ramachandran, 1965), electrostatically neutral, and balanced in hydrogen‐bond donors and acceptors. Protein secondary structures internally satisfy backbone hydrogen‐bond donors and acceptors (Nelson et al., 2021), and side chains largely shield the peptide backbone from solvent. Proteins can form globular structures that exclude water.

By contrast, the RNA backbone is conformationally flexible (Hsiao et al., 2006), polyanionic, and dominated by hydrogen‐bond acceptors (Hershkovitz et al., 2003; Murthy et al., 1999). The side chains (nucleobases) do not shield the RNA backbone from solvent. The polyanionic phosphodiester backbone lies on the exterior of secondary elements, is highly hydrated, and interacts with stabilizing counterions such as K^+^ and Mg^2+^. RNA folds into globular structures permeated by water and ions.

**FIGURE 5:**
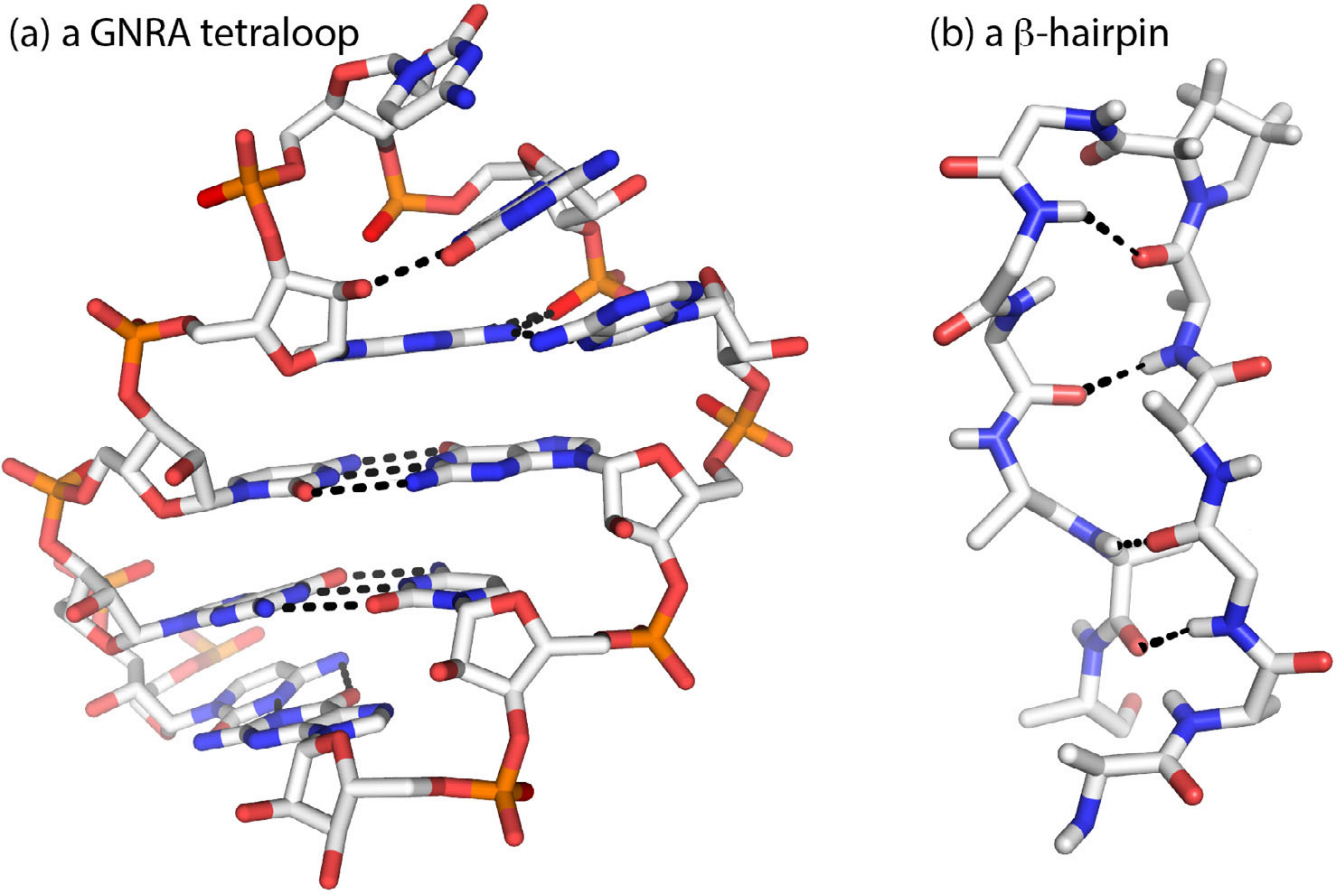
Acute strand reversals of biopolymers. (a) An RNA GNRA tetraloop. (b) A protein *β*‐turn in a *β*‐hairpin.

In spite of these distinctions, the three‐dimensional architecture of both polymers obeys common geometric imperatives. The topography of both folded proteins and structured RNAs consists of linear segments (e.g., helices) connected by turns and loops that reverse the overall direction of the backbone chain (Brion & Westhof, 1997; Rose et al., 1985). In both polymers, tight turns form cross strand hydrogen bonds (Figure 5) that bring the strands into proximity.

In hierarchical systems such as biopolymers (Brion & Westhof, 1997; Rose, 1979), local order (secondary structure) arises first, converting chains into series of rigid rods. Compactness then requires hinge points—tight, solvent‐exposed turns and loops—that reverse direction while preserving local order (Rose, 2025). Their necessity follows from the quasi‐independence of secondary and tertiary structure formation. The architectural convergence of protein and RNA thus reflects geometry and hierarchy: a long chain that folds hierarchically into a compact particle must incorporate localized reversals connecting its ordered segments. This hierarchical organization distinguishes folding from winding.

Tight turns and hairpins are the minimal, stereochemically regular devices for accomplishing these reversals while keeping local order (helices) intact (Rose et al., 1985). Hence, both proteins and RNAs employ frequent turns to bring linear segments into proximity and achieve globularity. These turns typically occur near the periphery of the folded structure (Kuntz, 1972), where they interact extensively with water and, in the case of RNA, with ions. Thus, although proteins are distinguished from RNAs by chemical asymmetries, both rely on tight, solvent‐exposed turns to achieve globularity.

A characteristic property of biopolymers is the propensity of subtle chemical changes to cause profound differences in structure, assembly, and function. Frequent prolines in a polypeptide inhibit the formation of *α*‐helices or *β*‐sheets, biasing the structure toward non‐catalytic collagen‐type assemblies (Shoulders & Raines, 2009). Conversion of polyalanine to polyglycine converts *α*‐helices to intrinsically disordered segments (Radivojac et al., 2007). RNA and DNA backbones differ by a single atom, but that difference changes the assembly states, helical form, hydrolytic lifetime, and catalytic potential (Nelson et al., 2021). If the anomeric linkage of polyglucose is changed from *β*(1,4) to *α*(1,4), the assembly state, hydrolytic lifetime, and function change dramatically. This minor chemical change converts cellulose (Habibi et al., 2010) to amylose (Fittolani et al., 2020; Roach et al., 2012). In each case, the functional consequences of these subtle chemical changes are ultimately expressed through altered interactions with water.

## 4 CONCLUSION

Thales of Miletus, a pre‐Socratic philosopher in the 7th century BCE, proposed that all nature originates from water (Kirk et al., 1983). In modern times, Lawrence Henderson was among the first to fully recognize the centrality of water as both a biological medium and a biochemical reactant and product (Henderson, 1913). Today, water is understood as an active and dynamic medium that governs broadly molecular behavior. It serves simultaneously as solvent, reaction substrate, reaction product, and energy currency, mediating the transfer of energy from the environment to organic molecules and among organic molecules.

The specific roles of water in biochemistry and its profound entanglement with both organic and inorganic species in modern biological systems suggest that prebiotic chemical evolution developed with water as a master molecule. Prior to the origins of life, Earth's land and oceans experienced day–night cycles, weather, and seasonal and climatic rhythms, as well as variations across latitude and local geography. A diverse ensemble of molecules and molecular assemblies predicted for the ancient Earth (Cody et al., 2000; Ehrenfreund & Sephton, 2006; Glavin et al., 2025; Parker et al., 2011; Wogan et al., 2024; Zahnle et al., 2020) was subject to the full range of physical and chemical behaviors expressed by water. We suggest that temporally and spatially variable environmental conditions promoted exploration of the state‐dependent properties of water and continuous sampling of water‐based chemistries. Molecules that could cooperate with, resist, or exploit the properties of water were selected and sculpted. Water served as a primary agent of selection, establishing a chemical framework for life's processes: polymerization, reactivity and recycling, assembly, recalcitrance and catalysis, and ultimately replication and Darwinian evolution. Our model of water as the master molecule suggests new experimental avenues for understanding life's emergence (Forsythe et al., 2015; Matange, Rajaei, et al., 2025; Ross & Deamer, 2016; Yu et al., 2016).

## AUTHOR CONTRIBUTIONS

**Juliana DiGiacomo:** Writing – review and editing; formal analysis; investigation. **George D. Rose:** Conceptualization; writing – original draft; methodology; software; validation; writing – review and editing; formal analysis; investigation. **Loren Dean Williams:** Conceptualization; writing – original draft; funding acquisition; methodology; validation; writing – review and editing; software; formal analysis; project administration; supervision; resources; investigation.

## Data Availability

All new data is contained in the paper.

## References

[pro70532-bib-0001] Alley R , Cuffey K , Zoet L . Glacial erosion: status and outlook. Ann Glaciol. 2019;60:1–13.

[pro70532-bib-0002] Anfinsen CB . Principles that govern the folding of protein chains. Science. 1973;181:223–230. 4124164 10.1126/science.181.4096.223

[pro70532-bib-0003] Baker EN , Hubbard RE . Hydrogen bonding in globular proteins. Prog Biophys Mol Biol. 1984;44:97–179. 6385134 10.1016/0079-6107(84)90007-5

[pro70532-bib-0004] Baldwin RL , Rose GD . How the hydrophobic factor drives protein folding. Proc Natl Acad Sci USA. 2016;113:12462–12466. 27791131 10.1073/pnas.1610541113 PMC5098675

[pro70532-bib-0005] Ball P . Water as an active constituent in cell biology. Chem Rev. 2008;108:74–108. 18095715 10.1021/cr068037a

[pro70532-bib-0006] Ball P . Water is an active matrix of life for cell and molecular biology. Proc Natl Acad Sci USA. 2017;114:13327–13335. 28592654 10.1073/pnas.1703781114 PMC5754758

[pro70532-bib-0007] Barbosa‐Cánovas GV , Fontana JAJ , Schmidt SJ , Labuza TP . Water activity in foods: fundamentals and applications. Hoboken, New Jersey: John Wiley & Sons; 2020.

[pro70532-bib-0008] Beckham GT , Matthews JF , Peters B , Bomble YJ , Himmel ME , Crowley MF . Molecular‐level origins of biomass recalcitrance: Decrystallization free energies for four common cellulose polymorphs. J Phys Chem B. 2011;115:4118–4127. 21425804 10.1021/jp1106394

[pro70532-bib-0009] Bellissima S , de Panfilis S , Bafile U , Cunsolo A , González MA , Guarini E , et al. The hydrogen‐bond collective dynamics in liquid methanol. Sci Rep. 2016;6:39533. 27996056 10.1038/srep39533 PMC5172242

[pro70532-bib-0010] Benner SA . Paradoxes in the origin of life. Orig Life Evol Biosph. 2014;44:339–343. 25608919 10.1007/s11084-014-9379-0

[pro70532-bib-0011] Bleuzen A , Pittet P‐A , Helm L , Merbach AE . Water exchange on magnesium(ii) in aqueous solution: a variable temperature and pressure 17o NME study. Magn Reson Chem. 1997;35:765–773.

[pro70532-bib-0012] Bolen DW , Rose GD . Structure and energetics of the hydrogen‐bonded backbone in protein folding. Annu Rev Biochem. 2008;77:339–362. 18518824 10.1146/annurev.biochem.77.061306.131357

[pro70532-bib-0013] Bowman JC , Lenz TK , Hud NV , Williams LD . Cations in charge: magnesium ions in RNA folding and catalysis. Curr Opin Struct Biol. 2012;22:262–272. 22595008 10.1016/j.sbi.2012.04.006

[pro70532-bib-0014] Brack A . Liquid water and the origin of life. Orig Life Evol Biosph. 1993;23:3–10. 11536525 10.1007/BF01581985

[pro70532-bib-0015] Brewer PG , Peltzer ET . The molecular basis for the heat capacity and thermal expansion of natural waters. Geophys Res Lett. 2019;46:13227–13233.

[pro70532-bib-0016] Brini E , Fennell CJ , Fernandez‐Serra M , Hribar‐Lee B , Lukšič M , Dill KA . How water's properties are encoded in its molecular structure and energies. Chem Rev. 2017;117:12385–12414. 28949513 10.1021/acs.chemrev.7b00259 PMC5639468

[pro70532-bib-0017] Brion P , Westhof E . Hierarchy and dynamics of RNA folding. Annu Rev Biophys Biomol Struct. 1997;26:113–137. 9241415 10.1146/annurev.biophys.26.1.113

[pro70532-bib-0018] Butler RN , Coyne AG . Water: Nature's reaction enforcer—comparative effects for organic synthesis “in‐water” and “on‐water”. Chem Rev. 2010;110:6302–6337. 20815348 10.1021/cr100162c

[pro70532-bib-0019] Campbell TD , Febrian R , Mccarthy JT , Kleinschmidt HE , Forsythe JG , Bracher PJ . Prebiotic condensation through wet–dry cycling regulated by deliquescence. Nat Commun. 2019;10:1–7. 31586058 10.1038/s41467-019-11834-1 PMC6778215

[pro70532-bib-0020] Ceccarelli C . Water in the universe. In: Gargaud M , Irvine WM , Amils R , Claeys P , Cleaves HJ , Gerin M , et al., editors. Encyclopedia of astrobiology. Berlin, Heidelberg: Springer; 2020. p. 1–5.

[pro70532-bib-0021] Cody GD , Boctor NZ , Filley TR , Hazen RM , Scott JH , Sharma A , et al. Primordial carbonylated iron‐sulfur compounds and the synthesis of pyruvate. Science. 2000;289:1337–1340. 10958777 10.1126/science.289.5483.1337

[pro70532-bib-0022] Creighton TE . Proteins: structures and molecular properties. New York, NY: W.H. Freeman & Co; 1993.

[pro70532-bib-0023] Damer B , Deamer D . Coupled phases and combinatorial selection in fluctuating hydrothermal pools: a scenario to guide experimental approaches to the origin of cellular life. Life. 2015;5:872–887. 25780958 10.3390/life5010872 PMC4390883

[pro70532-bib-0024] Damer B , Deamer D . The hot spring hypothesis for an origin of life. Astrobiology. 2020;20:429–452. 31841362 10.1089/ast.2019.2045 PMC7133448

[pro70532-bib-0025] Deamer D , Weber AL . Bioenergetics and life's origins. Cold Spring Harb Perspect Biol. 2010;2:a004929. 20182625 10.1101/cshperspect.a004929 PMC2828274

[pro70532-bib-0026] Dill KA . Dominant forces in protein folding. Biochemistry. 1990;29:7133–7155. 2207096 10.1021/bi00483a001

[pro70532-bib-0027] Dudev T , Lim C . Competition among metal ions for protein binding sites: determinants of metal ion selectivity in proteins. Chem Rev. 2014;114:538–556. 24040963 10.1021/cr4004665

[pro70532-bib-0028] Edri R , Fisher S , Menor‐Salvan C , Williams LD , Frenkel‐Pinter M . Assembly‐driven protection from hydrolysis as key selective force during chemical evolution. FEBS Lett. 2023;597:2879–2896. 37884438 10.1002/1873-3468.14766

[pro70532-bib-0029] Ehrenfreund P , Sephton MA . Carbon molecules in space: from astrochemistry to astrobiology. Faraday Discuss. 2006;133:277–288. 17191452 10.1039/b517676j

[pro70532-bib-0030] Erastova V , Degiacomi MT , G. Fraser D , Greenwell HC . Mineral surface chemistry control for origin of prebiotic peptides. Nat Commun. 2017;8:2033. 29229963 10.1038/s41467-017-02248-y PMC5725419

[pro70532-bib-0031] Faraday M . I. Note on regelation. Proc R Soc Lond. 1860;10:440–450. 10.1098/rspl.1859.0082

[pro70532-bib-0032] Ferris JP . Montmorillonite‐catalysed formation of RNA oligomers: the possible role of catalysis in the origins of life. Philos Trans R Soc Lond B Biol Sci. 2006;361:1777–1786. 17008218 10.1098/rstb.2006.1903 PMC1664692

[pro70532-bib-0033] Fersht A . Enzyme structure and mechanism. 2nd ed. New York: W. H. Freeman and Co; 1985.

[pro70532-bib-0034] Finney JL . Water? What's so special about it? Philos Trans R Soc Lond B Biol Sci. 2004;359:1145–1165. 15306373 10.1098/rstb.2004.1495 PMC1693413

[pro70532-bib-0035] Fittolani G , Seeberger PH , Delbianco M . Helical polysaccharides. Pept Sci. 2020;112:e24124.

[pro70532-bib-0036] Forsythe JG , Yu SS , Mamajanov I , Grover MA , Krishnamurthy R , Fernandez FM , et al. Ester‐mediated amide bond formation driven by wet‐dry cycles: a possible path to polypeptides on the prebiotic earth. Angew Chem Int Ed. 2015;54:9871–9875. 10.1002/anie.201503792 PMC4678426 26201989

[pro70532-bib-0037] Frenkel‐Pinter M , Bouza M , Fernández FM , Leman LJ , Williams LD , Hud NV , et al. Thioesters provide a plausible prebiotic path to proto‐peptides. Nat Commun. 2022;13:1–8. 35562173 10.1038/s41467-022-30191-0 PMC9095695

[pro70532-bib-0038] Frenkel‐Pinter M , Rajaei V , Glass JB , Hud NV , Williams LD . Water and life: the medium is the message. J Mol Evol. 2021;89:1–10. 33427903 10.1007/s00239-020-09978-6 PMC7884305

[pro70532-bib-0039] Glavin DP , Dworkin JP , Alexander CMO , Aponte JC , Baczynski AA , Barnes JJ , et al. Abundant ammonia and nitrogen‐rich soluble organic matter in samples from asteroid (101955) bennu. Nat Astron. 2025;9:199–210. 39990238 10.1038/s41550-024-02472-9 PMC11842271

[pro70532-bib-0040] Goldschmidt VM . Geochemistry. Oxford: Clarendon Press; 1954.

[pro70532-bib-0041] Graziano G . The cost of cavity creation depends on geometry. J Mol Liq. 2015;211:1047–1051.

[pro70532-bib-0042] Guardia E , Skarmoutsos I , Masia M . Hydrogen bonding and related properties in liquid water: a car–parrinello molecular dynamics simulation study. J Phys Chem B. 2015;119:8926–8938. 25313871 10.1021/jp507196q

[pro70532-bib-0043] Guth‐Metzler R , Mohamed AM , Cowan ET , Henning A , Ito C , Frenkel‐Pinter M , et al. Goldilocks and rna: where mg^2+^ concentration is just right. Nucleic Acids Res. 2023;51:3529–3539. 36987860 10.1093/nar/gkad124 PMC10164553

[pro70532-bib-0044] Habibi Y , Lucia LA , Rojas OJ . Cellulose nanocrystals: chemistry, self‐assembly, and applications. Chem Rev. 2010;110:3479–3500. 20201500 10.1021/cr900339w

[pro70532-bib-0045] Hagler A , Huler E , Lifson S . Energy functions for peptides and proteins. I. Derivation of a consistent force field including the hydrogen bond from amide crystals. J Am Chem Soc. 1974;96:5319–5327. 4851860 10.1021/ja00824a004

[pro70532-bib-0046] Hagler A , Lifson S . Energy functions for peptides and proteins. Ii. Amide hydrogen bond and calculation of amide crystal properties. J Am Chem Soc. 1974;96:5327–5335. 4851861 10.1021/ja00824a005

[pro70532-bib-0047] Hassanali A , Giberti F , Cuny J , Kühne TD , Parrinello M . Proton transfer through the water gossamer. Proc Natl Acad Sci USA. 2013;110:13723–13728. 23868853 10.1073/pnas.1306642110 PMC3752248

[pro70532-bib-0048] Hatters DM . Grand challenges in biomolecular condensates: structure, function, and formation. Front Biophys. 2023;1:1208763.

[pro70532-bib-0049] Hazen RM , Sverjensky DA . Mineral surfaces, geochemical complexities, and the origins of life. Cold Spring Harb Perspect Biol. 2010;2:a002162. 20452963 10.1101/cshperspect.a002162 PMC2857174

[pro70532-bib-0050] Held C , Cameretti LF , Sadowski G . Measuring and modeling activity coefficients in aqueous amino‐acid solutions. Ind Eng Chem Res. 2011;50:131–141.

[pro70532-bib-0051] Helm L , Merbach AE . Inorganic and bioinorganic solvent exchange mechanisms. Chem Rev. 2005;105:1923–1960. 15941206 10.1021/cr030726o

[pro70532-bib-0052] Henderson LJ . The fitness of the environment, an inquiry into the biological significance of the properties of matter. Am Nat. 1913;47:105–115.

[pro70532-bib-0053] Hershkovitz E , Tannenbaum E , Howerton SB , Sheth A , Tannenbaum A , Williams LD . Automated identification of RNA conformational motifs: theory and application to the HM LSU 23s RNA. Nucleic Acids Res. 2003;31:6249–6257. 14576313 10.1093/nar/gkg835 PMC275477

[pro70532-bib-0054] Hill A , Orgel L . Trimetaphosphate‐induced addition of aspartic acid to oligo(glutamic acid)s. Helv Chim Acta. 2002;85:4111–4578.

[pro70532-bib-0055] Hsiao C , Mohan S , Hershkovitz E , Tannenbaum A , Williams LD . Single nucleotide RNA choreography. Nucleic Acids Res. 2006;34:1481–1491. 16531589 10.1093/nar/gkj500 PMC1401506

[pro70532-bib-0056] Hsiao C , Williams LD . A recurrent magnesium‐binding motif provides a framework for the ribosomal peptidyl transferase center. Nucleic Acids Res. 2009;37:3134–3142. 19279186 10.1093/nar/gkp119 PMC2691814

[pro70532-bib-0057] Imai E‐I , Honda H , Hatori K , Brack A , Matsuno K . Elongation of oligopeptides in a simulated submarine hydrothermal system. Science. 1999;283:831–833. 9933163 10.1126/science.283.5403.831

[pro70532-bib-0058] Iogansen AV . Direct proportionality of the hydrogen bonding energy and the intensification of the stretching ν(xh) vibration in infrared spectra. Spectrochim Acta A Mol Biomol Spectrosc. 1999;55:1585–1612.

[pro70532-bib-0059] Jeffrey GA , Saenger W . Hydrogen bonding in biological structures. Berlin, Heidelberg: Springer Science & Business Media; 2012.

[pro70532-bib-0060] Kauzmann W . Some factors in the interpretation of protein denaturation. Adv Protein Chem. 1959;14:1–63. 14404936 10.1016/s0065-3233(08)60608-7

[pro70532-bib-0061] Kirk GS , Raven JE , Schofield M . The presocratic philosophers: a critical history with a selection of texts. Cambridge, England: Cambridge University Press; 1983.

[pro70532-bib-0062] Klein C , Dutrow B . Manual of mineral science. Hoboken, NJ: John Wiley & Sons; 2007.

[pro70532-bib-0063] Knight C , Voth GA . The curious case of the hydrated proton. Acc Chem Res. 2012;45:101–109. 21859071 10.1021/ar200140h

[pro70532-bib-0064] Korenaga J . Was there land on the early earth? Life. 2021;11:1142. 34833018 10.3390/life11111142 PMC8623345

[pro70532-bib-0065] Krishna SS , Majumdar I , Grishin NV . Structural classification of zinc fingers: survey and summary. Nucleic Acids Res. 2003;31:532–550. 12527760 10.1093/nar/gkg161 PMC140525

[pro70532-bib-0066] Krishnamoorthy A , Nomura K‐I , Baradwaj N , Shimamura K , Ma R , Fukushima S , et al. Hydrogen bonding in liquid ammonia. J Phys Chem Lett. 2022;13:7051–7057. 35900140 10.1021/acs.jpclett.2c01608 PMC9358710

[pro70532-bib-0067] Kuntz ID . Protein folding. J Am Chem Soc. 1972;94:4009–4012. 5037986 10.1021/ja00766a060

[pro70532-bib-0068] Lanier KA , Petrov AS , Williams LD . The central symbiosis of molecular biology: molecules in mutualism. J Mol Evol. 2017;85:8–13. 28785970 10.1007/s00239-017-9804-x PMC5579163

[pro70532-bib-0069] Lee B . Solvent reorganization contribution to the transfer thermodynamics of small nonpolar molecules. Biopolymers. 1991;31:993–1008. 1782360 10.1002/bip.360310809

[pro70532-bib-0070] Lee D , Redfern O , Orengo C . Predicting protein function from sequence and structure. Nat Rev Mol Cell Biol. 2007;8:995–1005. 18037900 10.1038/nrm2281

[pro70532-bib-0071] Lindahl T . Instability and decay of the primary structure of DNA. Nature. 1993;362:709–715. 8469282 10.1038/362709a0

[pro70532-bib-0072] Lipfert J , Doniach S , Das R , Herschlag D . Understanding nucleic acid‐ion interactions. Annu Rev Biochem. 2014;83:813–841. 24606136 10.1146/annurev-biochem-060409-092720 PMC4384882

[pro70532-bib-0073] Lippard SJ , Berg JM . Principles of bioinorganic chemistry. Mill Valley, CA: University Science Books; 1994.

[pro70532-bib-0074] Manning GS . Electrostatic free energy of the DNA double helix in counterion condensation theory. Biophys Chem. 2002;101:461–473. 12488020 10.1016/s0301-4622(02)00162-x

[pro70532-bib-0075] Marqusee S , Robbins VH , Baldwin RL . Unusually stable helix formation in short alanine‐based peptides. Proc Natl Acad Sci USA. 1989;86:5286–5290. 2748584 10.1073/pnas.86.14.5286 PMC297606

[pro70532-bib-0076] Martin RB . Free energies and equilibria of peptide bond hydrolysis and formation. Biopolymers. 1998;45:351–353.

[pro70532-bib-0077] Matange K , Marland E , Frenkel‐Pinter M , Williams LD . Biological polymers: evolution, function, and significance. Acc Chem Res. 2025;58:3137–3610. 39905926 10.1021/acs.accounts.4c00546 PMC11883738

[pro70532-bib-0078] Matange K , Rajaei V , Capera‐Aragones P , Costner JT , Robertson A , Kim JS , et al. Evolution of complex chemical mixtures reveals combinatorial compression and population synchronicity. Nat Chem. 2025;17:1–8. 39939341 10.1038/s41557-025-01734-x

[pro70532-bib-0079] Mauer L , Smith D , Labuza T . Effect of water content, temperature and storage on the glass transition, moisture sorption characteristics and stickiness of *β*‐casein. Int J Food Prop. 2000;3:233–248.

[pro70532-bib-0080] Menon G , Okeke C , Krishnan J . Modelling compartmentalization towards elucidation and engineering of spatial organization in biochemical pathways. Sci Rep. 2017;7:12057. 28935941 10.1038/s41598-017-11081-8 PMC5608717

[pro70532-bib-0081] Milo R , Phillips R . Cell biology by the numbers. New York, NY: Garland Science; 2015.

[pro70532-bib-0082] Murthy VL , Rose GD . Is counterion delocalization responsible for collapse in RNA folding? Biochemistry. 2000;39:14365–14370. 11087388 10.1021/bi001820r

[pro70532-bib-0083] Murthy VL , Srinivasan R , Draper DE , Rose GD . A complete conformational map for RNA. J Mol Biol. 1999;291:313–327. 10438623 10.1006/jmbi.1999.2958

[pro70532-bib-0084] Nelson DL , Lehninger AL , Cox MM . Lehninger principles of biochemistry. 8th ed. New York, NY: Macmillan; 2021.

[pro70532-bib-0085] Omont A . Molecules in galaxies. Rep Prog Phys. 2007;70:1099.

[pro70532-bib-0086] Orabi EA , Faraldo‐Gómez JD . New molecular‐mechanics model for simulations of hydrogen fluoride in chemistry and biology. J Chem Theory Comput. 2020;16:5105–5126. 32615034 10.1021/acs.jctc.0c00247 PMC8431972

[pro70532-bib-0087] Orgel LE . Polymerization on the rocks: theoretical introduction. Orig Life Evol Biosph. 1998;28:227–234. 9611763 10.1023/a:1006595411403

[pro70532-bib-0088] Ozkanlar A . Structural properties of hydrogen‐bond network in liquid formamide‐water mixtures. Fluid Phase Equilib. 2018;456:98–108.

[pro70532-bib-0089] Parker ET , Cleaves HJ , Bada JL , Fernández FM , Mathis DG . Enhanced hydrogen cyanide production from electrical discharge in neutral planetary atmospheres. Proc Natl Acad Sci USA. 2011;108:5526–5531.21422282

[pro70532-bib-0090] Peller L . On the free‐energy changes in the synthesis and degradation of nucleic acids. Biochemistry. 1976;15:141–146. 1247501 10.1021/bi00646a021

[pro70532-bib-0091] Phan TB , Mayr H . Comparison of the nucleophilicities of alcohols and alkoxides. Can J Chem. 2005;83:1554–1560.

[pro70532-bib-0092] Pimentel GC , Mcclellan A . Hydrogen bonding. Annu Rev Phys Chem. 1971;22:347–385.

[pro70532-bib-0093] Powner MW , Gerland B , Sutherland JD . Synthesis of activated pyrimidine ribonucleotides in prebiotically plausible conditions. Nature. 2009;459:239–242. 19444213 10.1038/nature08013

[pro70532-bib-0094] Prywes N , Blain JC , del Frate F , Szostak JW . Nonenzymatic copying of RNA templates containing all four letters is catalyzed by activated oligonucleotides. Elife. 2016;5:e17756. 27351102 10.7554/eLife.17756 PMC4959843

[pro70532-bib-0095] Rabinowitz J , Flores J , Kresbach R , Rogers G . Peptide formation in the presence of linear or cyclic polyphosphates. Nature. 1969;224:795–796. 4188388 10.1038/224795a0

[pro70532-bib-0096] Radivojac P , Iakoucheva LM , Oldfield CJ , Obradovic Z , Uversky VN , Dunker AK . Intrinsic disorder and functional proteomics. Biophys J. 2007;92:1439–1456. 17158572 10.1529/biophysj.106.094045 PMC1796814

[pro70532-bib-0097] Ramakrishnan C , Ramachandran G . Stereochemical criteria for polypeptide and protein chain conformations: II. Allowed conformations for a pair of peptide units. Biophys J. 1965;5:909–933. 5884016 10.1016/S0006-3495(65)86759-5 PMC1367910

[pro70532-bib-0098] Roach PJ , Depaoli‐Roach AA , Hurley TD , Tagliabracci VS . Glycogen and its metabolism: some new developments and old themes. Biochem J. 2012;441:763–787. 22248338 10.1042/BJ20111416 PMC4945249

[pro70532-bib-0099] Rose GD . Hierarchic organization of domains in globular proteins. J Mol Biol. 1979;134:447–470. 537072 10.1016/0022-2836(79)90363-2

[pro70532-bib-0100] Rose GD . Protein folding‐seeing is deceiving. Protein Sci. 2021;30:1606–1616. 33938055 10.1002/pro.4096 PMC8284583

[pro70532-bib-0101] Rose GD . From propensities to patterns to principles in protein folding. Proteins Struct Funct Bioinform. 2025;93:105–111. 10.1002/prot.26540 37353953

[pro70532-bib-0102] Rose GD , Fleming PJ , Banavar JR , Maritan A . A backbone‐based theory of protein folding. Proc Natl Acad Sci USA. 2006;103:16623–16633. 17075053 10.1073/pnas.0606843103 PMC1636505

[pro70532-bib-0103] Rose GD , Glerasch LM , Smith JA . Turns in peptides and proteins. Adv Protein Chem. 1985;37:1–109. 2865874 10.1016/s0065-3233(08)60063-7

[pro70532-bib-0104] Rose GD , Wolfenden R . Hydrogen bonding, hydrophobicity, packing, and protein folding. Annu Rev Biophys Biomol Struct. 1993;22:381–415. 8347995 10.1146/annurev.bb.22.060193.002121

[pro70532-bib-0105] Ross D , Deamer D . Dry/wet cycling and the thermodynamics and kinetics of prebiotic polymer synthesis. Life. 2016;6:28. 27472365 10.3390/life6030028 PMC5041004

[pro70532-bib-0106] Ross D , Deamer D . Prebiotic oligomer assembly: what was the energy source? Astrobiology. 2019;19:517–521. 30707599 10.1089/ast.2018.1918

[pro70532-bib-0107] Sarkar S , Bandyopadhyay B . Cooperative nature of the sulfur centered hydrogen bond: investigation of (h_2_s)_n_ (*n* = 2–4) clusters using an affordable yet accurate level of theory. Phys Chem Chem Phys. 2019;21:25439–25448. 31712792 10.1039/c9cp05326c

[pro70532-bib-0108] Scholtz JM , Marqusee S , Baldwin RL , York EJ , Stewart JM , Santoro M , et al. Calorimetric determination of the enthalpy change for the alpha‐helix to coil transition of an alanine peptide in water. Proc Natl Acad Sci USA. 1991;88:2854–2858. 2011594 10.1073/pnas.88.7.2854 PMC51338

[pro70532-bib-0109] Senthilkumar L , Ghanty TK , Ghosh SK , Kolandaivel P . Hydrogen bonding in substituted formic acid dimers. J Phys Chem A. 2006;110:12623–12628. 17107113 10.1021/jp061285q

[pro70532-bib-0110] Shapiro R . Origins: a skeptic's guide to the creation of life on earth. New York: Summit Books; 1986.

[pro70532-bib-0111] Shoulders MD , Raines RT . Collagen structure and stability. Annu Rev Biochem. 2009;78:929–958. 19344236 10.1146/annurev.biochem.77.032207.120833 PMC2846778

[pro70532-bib-0112] Slade L , Levine H , Reid DS . Beyond water activity: recent advances based on an alternative approach to the assessment of food quality and safety. Crit Rev Food Sci Nutr. 1991;30:115–360. 1854434 10.1080/10408399109527543

[pro70532-bib-0113] Steinman G , Lemmon RM , Calvin M . Cyanamide: a possible key compound in chemical evolution. Proc Natl Acad Sci USA. 1964;52:27–30. 14192655 10.1073/pnas.52.1.27 PMC300564

[pro70532-bib-0114] Stickle DF , Presta LG , Dill KA , Rose GD . Hydrogen bonding in globular proteins. J Mol Biol. 1992;226:1143–1159. 1518048 10.1016/0022-2836(92)91058-w

[pro70532-bib-0115] Sutherland JD . Opinion: studies on the origin of life—the end of the beginning. Nat Rev Chem. 2017;1:0012.

[pro70532-bib-0116] Tanford C . Contribution of hydrophobic interactions to the stability of the globular conformation of proteins. J Am Chem Soc. 1962;84:4240–4247.

[pro70532-bib-0117] Tanford C . The hydrophobic effect: formation of micelles and biological membranes. 2nd ed. New York, NY: (J. Wiley); 1980.

[pro70532-bib-0118] Taylor R , Kennard O , Versichel W . Geometry of the imino‐carbonyl (nh… O:C) hydrogen bond. 1. Lone‐pair directionality. J Am Chem Soc. 1983;105:5761–5766.

[pro70532-bib-0119] von Hippel PH , Berg OG . On the specificity of DNA‐protein interactions. Proc Natl Acad Sci USA. 1986;83:1608–1612. 3456604 10.1073/pnas.83.6.1608 PMC323132

[pro70532-bib-0120] von Hippel PH , Schleich T . Ion effects on the solution structure of biological macromolecules. Acc Chem Res. 1969;2:257–265.

[pro70532-bib-0121] Wächtershäuser G . Before enzymes and templates: theory of surface metabolism. Microbiol Rev. 1988;52:452–484. 3070320 10.1128/mr.52.4.452-484.1988 PMC373159

[pro70532-bib-0122] Weller M , Rourke J , Armstrong F , Lancaster S , Overton T . Inorganic chemistry. 8th ed. USA: Oxford University Press; 2025.

[pro70532-bib-0123] Westheimer FH . Why nature chose phosphates. Science. 1987;235:1173–1178. 2434996 10.1126/science.2434996

[pro70532-bib-0124] Wogan NF , Catling DC , Zahnle KJ . Timing and likelihood of the origin of life derived from post‐impact highly reducing atmospheres. Astrobiology. 2024;24:881–891. 39344973 10.1089/ast.2023.0128

[pro70532-bib-0125] Wolfenden R . Degrees of difficulty of water‐consuming reactions in the absence of enzymes. Chem Rev. 2006;106:3379–3396. 16895333 10.1021/cr050311y

[pro70532-bib-0126] Wolfenden R , Lu X , Young G . Spontaneous hydrolysis of glycosides. J Am Chem Soc. 1998;120:6814–6815.

[pro70532-bib-0127] Wright MR . An introduction to aqueous electrolyte solutions. Hoboken, New Jersey: John Wiley & Sons; 2007.

[pro70532-bib-0128] Yan S , Gawlak G , Makabe K , Tereshko V , Koide A , Koide S . Hydrophobic surface burial is the major stability determinant of a flat, single‐layer *β*‐sheet. J Mol Biol. 2007;368:230–243. 17335845 10.1016/j.jmb.2007.02.003 PMC1995161

[pro70532-bib-0129] Yu SS , Krishnamurthy R , Fernandez FM , Hud NV , Schork FJ , Grover MA . Kinetics of prebiotic depsipeptide formation from the ester‐amide exchange reaction. Phys Chem Chem Phys. 2016;18:28441–28450. 27711571 10.1039/c6cp05527c

[pro70532-bib-0130] Zahnle KJ , Lupu R , Catling DC , Wogan N . Creation and evolution of impact‐generated reduced atmospheres of early earth. Planet Sci J. 2020;1:11.

[pro70532-bib-0131] Zhang S , Lockshin C , Cook R , Rich A . Unusually stable *β*‐sheet formation in an ionic self‐complementary oligopeptide. Biopolymers. 1994;34:663–672. 8003624 10.1002/bip.360340508

